# Sociodemographic Factors, Physical Activity and Glycemic Control in Adults with Diabetes: A Pilot Study from a Croatian Reference Center

**DOI:** 10.3390/nursrep15110408

**Published:** 2025-11-19

**Authors:** Irena Canjuga, Dijana Vuković, Vilma Kolarić, Dario Rahelić, Goran Kozina, Vesna Mijoč, Melita Sajko, Natalija Uršulin-Trstenjak, Mihaela Kranjčević Ščurić, Iva Lončarić Kelečić

**Affiliations:** 1 Department of Nursing, University North, 104. Brigade 3, 42000 Varaždin, Croatia; melita.sajko@unin.hr (M.S.); mihaela.kranjcevic-scuric@unin.hr (M.K.Š.); 2 Department of Public Relations, University North, 104. Brigade 3, 42000 Varaždin, Croatia; dijana.vukovic@unin.hr; 3 Merkur Clinical Hospital, Vuk Vrhovac University Clinic for Diabetes, Endocrinology and Metabolic Diseases, 10000 Zagreb, Croatia; vilma.kolaric@kb-merkur.hr (V.K.); dario.rahelic@gmail.com (D.R.); 4 Faculty of Health Studies, Catholic University of Croatia, 10000 Zagreb, Croatia; vesna.mijoc@unicath.hr; 5 Department of Economic, University North, 104. Brigade 3, 42000 Varaždin, Croatia; goran.kozina@unin.hr; 6 Department of Food Technology, University North, Trg dr. Žarka Dolinara 1, 48000 Koprivnica, Croatia; natalija.ursulin-trstenjak@unin.hr; 7 Department for Physical Therapy, University Clinical Hospital Centre Zagreb, 10000 Zagreb, Croatia; 8 Faculty of Health Sciences, Libertas International University, 10000 Zagreb, Croatia

**Keywords:** comorbidities, diabetes mellitus, glycemic control, health education, hemoglobin A1c (HbA1c), physical activity, sociodemographic factors, strength training

## Abstract

**Background/Objectives:** Diabetes mellitus (DM) is a major global health concern, yet limited research has examined how sociodemographic factors and physical activity (PA) influence glycaemic control within specific national contexts. This pilot study explored associations between sociodemographic and behavioral factors and glycaemic regulation among adults with DM in Croatia. **Methods:** A cross-sectional study was conducted at a national reference center, including 95 adults with type 1 or type 2 diabetes. Data on demographics, clinical characteristics, and PA were obtained through questionnaires and medical records. Descriptive statistics, Welch’s *t*-tests, χ^2^ tests, correlations, and regression analyses were applied to identify predictors of HbA1c and diabetes-related complications. Glycaemic control was categorized as optimal (HbA1c ≤ 7.5%) or suboptimal (>7.5%) according to the pragmatic clinical threshold commonly used in DM management. **Results:** Mean HbA1c was 6.9% (SD = 1.3), with 33.7% of participants above 7.5%. Higher education (β = −0.48, *p* = 0.013) and participation in strength or balance exercises (β = −0.32, *p* = 0.041) were associated with lower HbA1c, whereas longer disease duration (β = 0.03, *p* = 0.004) and type 2 diabetes (β = 0.38, *p* = 0.030) predicted higher HbA1c. In logistic regression, age predicted cardiovascular comorbidities (OR = 1.12, 95% CI 1.02–1.23, *p* = 0.019). The interaction between PA and place of residence (urban vs. rural) showed a non-significant trend (*p* = 0.061). **Conclusions:** Glycaemic control in Croatian adults with diabetes was associated with educational level and engagement in strength and balance exercises, while longer disease duration, older age, and type 2 diabetes were linked to poorer regulation and more complications. These findings underscore the importance of structured exercise and patient education in diabetes management, although larger, prospective studies with standardized PA-intensity measures are required to confirm and extend these results.

## 1. Introduction

Over the past three decades, the global prevalence of diabetes mellitus (DM) and impaired glucose tolerance (IGT) has quadrupled [1], making DM a major public health concern that affects approximately one in ten adults aged 20–79 years and ranks among the leading causes of premature mortality worldwide [2]. The number of cases is projected to reach 783.2 million by 2045 [3].

In Croatia, national data reflect this global trend: 396.005 diagnosed cases were recorded in 2024, although the actual number is likely to exceed half a million due to underdiagnosis [4]. Type 2 diabetes (T2DM) is primarily linked to modifiable lifestyle factors, particularly insufficient physical activity and poor dietary habits [5], alongside genetic predisposition and family history—approximately 60% of patients report at least one affected parent [6]. In contrast, type 1 diabetes (T1DM) is sporadic primarily, with 90% of cases lacking a positive family history [7]. Specific populations, such as women with polycystic ovary syndrome, are more susceptible to early-onset T2DM [5,8].

Chronic complications, including cardiovascular disease, neuropathy, diabetic foot, and retinopathy [9,10,11], as well as increased cancer risk [12,13], pose major health and socioeconomic burdens. On average, life expectancy is reduced by about six years in individuals with diabetes [14], who are further burdened by multiple comorbidities [15].

Glycated hemoglobin (HbA1c) is a well-established marker of long-term glycemic control, preferred over fasting glucose and oral glucose tolerance testing due to its practicality and stability [16]. Accordingly, epidemiological surveillance and clinical monitoring rely on HbA1c as a key indicator of metabolic regulation [17].

Numerous risk factors for T2DM have been identified, including age [18], obesity [19,20], gender [5], ethnicity [21], inactivity [22,23], poor diet [15,24], family history [6], gestational diabetes [25,26], hypertension [27], and dyslipidemia [28].

However, despite extensive global evidence on exercise and metabolic health, country-specific data on physical activity patterns and their association with glycemic control remain scarce in Central and Eastern Europe, including Croatia. Local differences in healthcare organization, health literacy, and cultural attitudes toward exercise may influence patients’ ability to meet international recommendations for physical activity. National and global diabetes strategies consistently emphasize lifestyle modification and structured exercise as central components of prevention and management. However, implementation and systematic monitoring at the population level remain limited [4]. Understanding these associations in the Croatian context may provide preliminary insights into behavioral and sociodemographic factors that influence glycemic regulation, serving as a foundation for future, larger-scale studies.

Therefore, the present pilot study aimed to investigate the relationships between sociodemographic, behavioral, and clinical factors and glycemic control among adults with diabetes. Specifically, the objectives were to: (1) describe the sociodemographic, clinical, and physical activity characteristics of the sample; (2) examine associations between physical activity patterns (type, frequency, and duration) and glycemic control (HbA1c levels); (3) identify predictors of glycemic control, including education, diabetes type, and disease duration; (4) explore the associations between glycemic regulation and diabetes-related complications, particularly cardiovascular comorbidities; and (5) identify potential behavioral or metabolic subgroups using cluster analysis.

We hypothesized that higher education and participation in strength and balance training would be associated with lower HbA1c levels, whereas longer disease duration, older age, and T2DM would predict poorer glycemic control and greater prevalence of complications.

## 2. Materials and Methods

In designing and conducting the study, efforts were made to prepare and align the methodology with the Strengthening the Reporting of Observational Studies in Epidemiology (STROBE) guidelines [29].

### 2.1. Participants and Procedure

This cross-sectional study was conducted over three months (December 2024 to February 2025) at the University Clinic for Diabetes, Endocrinology, and Metabolic Diseases Vuk Vrhovac, Merkur University Hospital in Zagreb, which also serves as the national Reference Center for Diabetes. Of 664 eligible patients attending the outpatient diabetes clinic during the study period, 95 consented to participate (response rate ≈ 14%). Reasons for non-participation included scheduling conflicts and personal preference (not systematically recorded). This exploratory single-center pilot study was intended to provide preliminary data and variance estimates to inform the design of a larger prospective multicentre study; therefore, both T1DM and T2DM were analyzed jointly to preserve statistical power.

Data were collected during routine appointments at the Center for Diabetes. Eligible participants were adults (≥18 years) with a confirmed diagnosis of DM (T1DM or T2DM). Individuals with a history of psychiatric illness or those who declined participation were excluded.

Participants were consecutively invited during their scheduled clinic visits and received both verbal and written information about the study aims, procedures, potential risks and benefits, and their right to withdraw at any time. Those who agreed to participate provided written informed consent.

Clinical parameters, including the most recent fasting glucose, HbA1c, total cholesterol, and serum creatinine values, were extracted from medical records. Participants independently completed a structured questionnaire covering sociodemographic characteristics, lifestyle factors, and self-reported physical activity. Assistance was provided when necessary, primarily to older participants experiencing visual difficulties or fatigue. Completion of the questionnaire required approximately 35 min on average.

All procedures were conducted in accordance with the ethical standards of the institutional research committee and the principles of the Declaration of Helsinki.

### 2.2. Instrument

Data were collected using a semi-structured, custom-developed questionnaire comprising three main sections. Participants reported their age, gender, education level, marital status, place of residence, employment status, height (m), and weight (kg). The questionnaire also included information on diabetes type (T1DM, T2DM, gestational, or secondary), disease duration (years), diabetes-related complications (e.g., myocardial infarction, stroke, arterial hypertension, diabetic foot, retinopathy, nephropathy, neuropathy), and type of therapy (none, oral medication, insulin, or combined therapy including GLP-1 receptor agonists or insulin pump). The most recent biochemical results: fasting glucose (mmol/L), HbA1c (%), serum creatinine (µmol/L), and total cholesterol (mmol/L), were extracted from medical records. Body mass index (BMI) was calculated as weight (kg) divided by height squared (m^2^).

Physical-activity patterns were assessed by type, frequency, and session duration. Participants reported engaging in aerobic activities (e.g., brisk walking, cycling, swimming, running, hiking, dancing), strength and resistance exercises (e.g., weight-lifting, use of fitness machines), and balance or proprioceptive activities (e.g., standing on one leg, tai chi).

Physical-activity frequency and session duration were self-reported and classified as low (<30 min), moderate (30–60 min), or high (60–120 min) per session, performed daily, twice per week, or three or more times per week. Because metabolic-equivalent (MET) data or physiological markers of exertion were unavailable, activity “intensity” was operationalised by session duration rather than actual physiological effort. In line with international recommendations [30,31], these categories therefore represent duration-based activity levels rather than standardized intensity levels.

Participants who exercised three or more times per week (totalling at least 120 min) were classified as “active,” following adult guidelines recommending at least 150 min of moderate-intensity or 75 min of vigorous-intensity activity weekly, while those who exercised two or fewer times per week or irregularly were classified as “less active.”

The questionnaire was developed specifically for this study and reviewed by a diabetes specialist, a physiologist, a nurse, and a physiotherapist to ensure content validity and clarity. This multidimensional instrument provided an exploratory assessment of physical activity behaviors and their potential associations with glycemic control and diabetes-related complications in this pilot sample.

### 2.3. Statistical Analysis

Statistical analyses were performed using IBM SPSS Statistics for Windows, Version 25.0 (IBM Corporation, Armonk, NY, USA). Normality was assessed using the Shapiro–Wilk test and quantile–quantile (Q–Q) plots [32]. Parametric tests were applied after verifying distributional assumptions. Descriptive statistics summarized sociodemographic, clinical, and physical activity variables.

Welch’s *t*-test was used to compare mean glycated hemoglobin (HbA1c) values between groups differing in physical activity levels, accounting for unequal variances and group sizes. Chi-square (χ^2^) tests assessed differences in the prevalence of chronic complications across glycemic control categories (HbA1c ≤ 7.5% vs. >7.5%), consistent with clinical practice thresholds commonly used to guide diabetes treatment pathways. Glycaemic control was classified as well-controlled when HbA1c ≤ 7.5% and uncontrolled when >7.5%. Although the 2023 American Diabetes Association and European Association for the Study of Diabetes consensus report recommends an HbA1c target of <7.0% for most adults [33], the 7.5% cutoff represents a pragmatic threshold commonly used in clinical practice to distinguish patients typically managed with metformin monotherapy (≤7.5%) from those requiring dual or intensified therapy (≥7.5%), and reflects the treatment-adjustment criterion applied in our setting.

Pearson’s correlation coefficient (r) was used to examine associations among continuous variables, such as disease duration, body mass index (BMI), and the number of complications. Predictors of glycemic control (HbA1c) were examined using simple and multiple linear regression models, including demographic, clinical, and lifestyle variables.

Categorical predictors were dummy-coded (gender: 0 = female, 1 = male; education: 0 = below university, 1 = university or higher; strength/balance training at least once per week: 0 = no, 1 = yes; diabetes type: 0 = type 1 diabetes mellitus (T1DM), 1 = type 2 diabetes mellitus (T2DM). Age and disease duration were entered as continuous variables. Initially, all covariates were entered in a full model; non-significant variables (*p* > 0.10) were sequentially removed to obtain a parsimonious model based on the Akaike Information Criterion (AIC) and adjusted coefficient of determination (adjusted R^2^).

Model assumptions for multiple linear regression: linearity, normality, homoscedasticity of residuals, and multicollinearity (variance inflation factor, VIF < 5) were verified [34,35]. Binary logistic regression was applied to identify predictors of participation in strength and balance training and cardiovascular comorbidities. Model fit was evaluated using the Hosmer–Lemeshow goodness-of-fit test, and discrimination was assessed using receiver operating characteristic/area under the curve (ROC/AUC) analysis [36].

Interaction terms were introduced to examine potential effect modification by place of residence (urban vs. rural) on the relationship between physical activity and HbA1c. To examine contextual influences, interaction terms were tested between physical-activity engagement (a composite variable combining self-reported frequency and session duration) and place of residence (urban vs. rural) in relation to HbA1c levels. This allowed the assessment of potential moderating effects of urbanicity on the relationship between physical activity and glycaemic control. K-means clustering (k = 3) was conducted to identify subgroups based on shared patterns in glycemic control, physical activity, and complications. The number of clusters was determined using the elbow criterion and verified for stability by silhouette analysis [37]; all variables were standardized (z-scores) prior to clustering.

Analyses were performed on complete cases; missing data per variable were <10% and not imputed. Given the exploratory nature of this pilot study, no adjustment for multiple comparisons was applied [38]. Statistical significance was set at *p* < 0.05 (two-tailed) for all analyses. This analytical approach provided an exploratory overview of relationships between sociodemographic, clinical, and behavioral factors associated with glycemic regulation in adults with diabetes.

## 3. Results

The final sample (Table 1) comprised 95 adults with diabetes, including 53 males (55.79%) and 42 females (44.21%). Although the study initially targeted a bimodal age structure to capture both younger and older adults, the final distribution was skewed toward older participants, with the largest proportions in the 65+ (n = 40; 42.11%) and 58–65 (n = 20; 21.05%) age groups. Younger age groups were progressively less represented, with the smallest proportion in the 26–33-year range (n = 3; 3.16%).

Regarding educational attainment, 56.84% (n = 54) reported secondary education as their highest level, 10.53% (n = 10) had a high school diploma, 14.74% (n = 14) had an undergraduate degree, and 32.63% (n = 31) had a graduate or higher degree.

In terms of marital status, 62.11% (n = 59) were married, 13.68% (n = 13) single, 12.63% (n = 12) widowed, and 5.26% (n = 5) divorced. Only 1.05% (n = 1) reported being in a relationship, engaged, or cohabiting, while 3.16% (n = 3) did not specify.

Most participants lived in urban areas (n = 74; 77.89%), and 22.11% (n = 21) resided in rural areas. Concerning employment status, 50.53% (n = 48) were retired, 43.16% (n = 41) employed full-time, 2.11% (n = 2) part-time, and 4.21% (n = 4) unemployed.

The clinical and lifestyle characteristics of the participants are presented in Table 2. Among the 95 adults with diabetes, 73.68% had T2DM (n = 70) and 26.32% had T1DM (n = 25). Consistent with this, 73.68% of participants received oral antidiabetic medication (n = 70), while 26.32% received insulin therapy (n = 25).

The most common comorbidity was arterial hypertension, reported by 63.16% of participants (n = 60). Other complications included retinopathy (31.58%; n = 30), diabetic foot (15.79%; n = 15), nephropathy (12.63%; n = 12), and neuropathy or polyneuropathy (8.42%; n = 8). Cardiovascular conditions such as myocardial infarction or stroke were present in 12.63% of participants (n = 12), while 10.53% (n = 10) reported other chronic conditions, including autoimmune and oncological diseases.

According to body mass index (BMI) classification, 28.72% of participants (n = 27) had normal weight, 42.55% (n = 40) were overweight, 17.02% (n = 16) were obese (BMI 30–34.99), 7.45% (n = 7) were severely obese (BMI 35–39.99), and 2.13% (n = 2) had morbid obesity (BMI ≥ 40).

Most participants reported engaging in aerobic physical activities, primarily walking or brisk walking (94.74%; n = 90), followed by cycling (21.05%; n = 20), swimming (15.79%; n = 15), hiking or Nordic walking (12.63%; n = 12), and dance, yoga, or Pilates (10.53%; n = 10). Anaerobic or strength training was less frequent, with 31.58% (n = 30) using elastic bands or body-weight exercises, 26.32% (n = 25) using free weights, and 21.05% (n = 20) using gym machines. Balance or coordination activities, such as one-leg stand or tai chi, were reported by 21.05% (n = 20) of participants.

Regarding exercise session duration, 26.3% (n = 25) of participants reported engaging in sessions lasting less than 30 min, 52.6% (n = 50) in 30–60 min sessions, and 21.1% (n = 20) in 60–120 min sessions. Regarding exercise frequency, 36.8% (n = 35) reported exercising daily, 42.1% (n = 40) three or more times per week, 15.8% (n = 15) twice per week, and 5.3% (n = 5) less than twice per week or irregularly.

To assess metabolic control and organ function in individuals with diabetes, descriptive statistics were calculated for four key laboratory biomarkers (Table 3): fasting glucose, glycated hemoglobin (HbA1c), total cholesterol, and serum creatinine. The average fasting glucose level was approximately 7.9 mmol/L (SD ≈ 2.3; range: 4.2–16.5 mmol/L) based on 82 participants. This distribution indicates substantial variability in short-term glycemic control, with some participants exhibiting normoglycemic values, while others demonstrated levels consistent with poorly managed hyperglycemia. The mean value for HbA1c, a key indicator of long-term glycemic regulation, was 6.9% (SD ≈ 1.3; range: 5.0–12.7%) across 90 participants. These results suggest that although a subset of the sample maintained satisfactory metabolic control (i.e., HbA1c ≤ 7.0%), a considerable proportion had elevated HbA1c levels, indicative of suboptimal disease management or progression. Regarding lipid metabolism, the mean total cholesterol concentration was approximately 4.6 mmol/L (SD ≈ 1.1; range: 2.1–7.8 mmol/L) in a subsample of 88 participants. This moderate mean value, alongside a broad range, reflects the co-existence of both normolipidemic and hypercholesterolemic profiles within the cohort. Lastly, the mean serum creatinine level was 76.8 µmol/L (SD ≈ 34.6; range: 0.7–204.0 µmol/L) in a sample of 81 individuals. The wide dispersion of values highlights substantial inter-individual differences in renal function, potentially including early-stage kidney impairment or advanced diabetic nephropathy in a subset of respondents.

These descriptive findings provide a clinically relevant overview of the sample’s metabolic and renal status, offering a basis for subsequent analyses examining associations with physical activity patterns, sociodemographic characteristics, and diabetes-related complications.

As shown in Table 4, participants with better glycemic control (HbA1c ≤ 7.5%) reported significantly higher weekly exercise frequency than those with elevated HbA1c (3.7 ± 0.8 vs. 3.1 ± 0.7; *p* = 0.015). When exercise session duration was examined, individuals with higher HbA1c were more likely to engage in shorter sessions (<30 min), with this difference reaching statistical significance (34.4% vs. 18.0%; *p* = 0.041). No significant between-group differences were observed for sessions lasting 30–60 min or 60–120 min (*p* = 0.372 and *p* = 0.229, respectively). Overall, the findings suggest that both greater weekly frequency and longer session duration are more common among individuals with controlled HbA1c, indicating a possible association between more sustained physical activity engagement and better glycemic outcomes.

**Figure 1:**
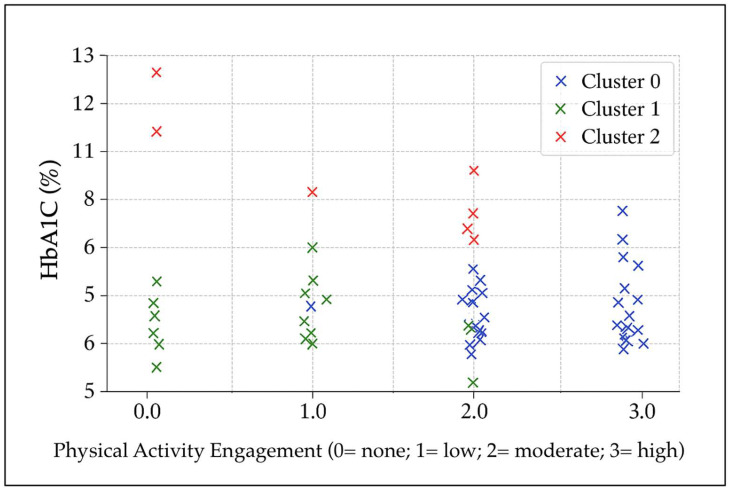
Two-dimensional projection of the three k-means clusters in the HbA1c–physical-activity engagement space. Note: derived from multivariate clustering of HbA1c, physical activity, and complications.

An exploratory k-means cluster analysis was conducted using glycated hemoglobin (HbA1c), the number of chronic diabetes-related complications, and physical-activity measures (session duration and frequency). Three distinct clusters emerged (Figure 1). Cluster 0 (n ≈ 46) comprised participants with well-controlled glycemia (mean HbA1c ≈ 6.6%) and higher physical-activity engagement, mainly exercising daily with sessions of moderate to long duration. The average number of complications was approximately 1, with 41% of participants reporting none. Cluster 1 (n ≈ 39) also showed favorable glycemic control (mean HbA1c ≈ 6.5%) but lower physical-activity engagement, typically exercising twice per week with short session durations or reporting no regular activity. Complications averaged around 1 per participant, with 38% reporting none. Cluster 2 (n ≈ 10) exhibited poor glycemic control (mean HbA1c ≈ 9.8%) despite engaging in moderate-frequency activity (3–4 days per week, sessions of low to moderate duration). This cluster had the lowest complication burden (mean ≈ 0.4), with 60% of participants reporting none, possibly reflecting shorter disease duration or early-stage diabetes. This group may therefore represent individuals at higher metabolic risk who require closer clinical monitoring and earlier intervention.

Table 5 presents the comparison of mean HbA1c values between participants classified as physically active (≥3 exercise sessions per week, typically of moderate or long duration) and those classified as less active. Welch’s *t*-test indicated no statistically significant difference between the groups, t (36) = −0.72, *p* = 0.479. The mean HbA1c value was slightly higher in the active group (6.86 ± 1.25%) than in the less active group (6.67 ± 1.01%), with a mean difference of 0.19 percentage points. However, the 95% confidence interval (−0.35 to 0.73) included zero, indicating that this difference was not statistically significant. The effect size was small (Cohen’s d = 0.16), suggesting a negligible difference in glycaemic control between groups.

These findings do not support the hypothesis that higher frequency or longer session duration of physical activity is associated with lower HbA1c levels in this sample.

The results presented in Table 6 show that elevated HbA1c levels (>7.5%) are significantly associated with hypertension (χ^2^ (1) = 6.79, *p* = 0.009; OR = 3.70, 95% CI [1.34–10.21]) and moderately associated with retinopathy (χ^2^ (1) = 5.23, *p* = 0.022; OR = 2.82, 95% CI [1.14–6.98]). In contrast, the association with nephropathy did not reach statistical significance (χ^2^ (1) = 1.64, *p* = 0.201; OR = 2.19, 95% CI [0.65–7.45]). The clinical implication is straightforward: maintaining HbA1c levels below 7.5% is not merely a numerical goal—it significantly reduces the odds of concurrent development of elevated blood pressure and visual impairment. These two complications critically impact the quality of life and long-term prognosis in individuals with diabetes.

As seen in Table 7, participants with a university degree or higher were 2.45 times more likely to engage in such exercise than those with lower levels of education (OR = 2.45, 95% CI [1.12–5.36], *p* = 0.025). Although age was not statistically significant (*p* = 0.110), the trend suggests a potential age-related barrier to participation.

Overall, the model highlights educational attainment as a key leverage point for interventions promoting structured physical activity, especially among populations at risk for diabetes-related complications.

The parsimonious logistic regression model (Table 7) retained no statistically significant predictors of engaging in strength and balance training at least once per week. Although the odds ratio for type 2 diabetes was notably high (OR = 26.51), the wide confidence interval (95% CI [0.03–24,329.90]) and non-significant *p*-value (*p* = 0.346) indicate a lack of statistical reliability. Other variables, including age, gender, and diabetes duration, also showed no significant associations with participation in strength and balance exercises.

The model suggests that, when controlling for these key clinical and demographic factors simultaneously, none is a strong independent predictor of engagement in this physical activity (Table 8). This outcome may reflect the complexity of factors influencing exercise behavior or insufficient statistical power due to sample size and variability.

The linear regression model identified several significant predictors of HbA1c levels, offering insight into both clinical and behavioral determinants of glycemic control (Table 9). Education level emerged as a significant and protective factor. Participants with a university-level education or higher had, on average, 0.48 percentage points lower HbA1c compared to those with lower education (β = −0.48, *p* = 0.013). This suggests that higher educational attainment may facilitate better diabetes self-management, possibly through improved health literacy, greater access to resources, or stronger adherence to treatment regimens. Engagement in strength and balance training was also significantly associated with improved glycemic control. Those who participated in such an exercise had an average HbA1c 0.32 percentage points lower (β = −0.32, *p* = 0.041). This finding highlights the potential benefits of incorporating resistance and neuromotor training, in addition to aerobic activity, into diabetes care, particularly given its accessibility and the additional advantages for musculoskeletal health and fall prevention. The duration of diabetes showed a small but statistically significant positive association with HbA1c (β = 0.03, *p* = 0.004), indicating that each additional year of diabetes was associated with a 0.03% increase in HbA1c. This trend is consistent with the known progressive nature of type 2 diabetes and the cumulative burden of disease, which may make glycemic control more difficult over time. Having T2DM (vs. T1DM) was also associated with higher HbA1c levels (β = 0.38, *p* = 0.030). While this may initially seem counterintuitive, it may reflect differences in treatment strategies, disease perception, or clinical inertia in managing T2DM compared to T1DM, which is more intensively managed. Age showed a marginal trend toward higher HbA1c with increasing years (β = 0.01, *p* = 0.080), though this did not reach statistical significance. This may indicate subtle age-related changes in metabolism, comorbidities, or treatment priorities (e.g., relaxed glycemic targets in older adults); however, further investigation would be necessary to confirm this. Gender (male) was not a significant predictor (β = −0.05, *p* = 0.730), suggesting no substantial gender-based difference in HbA1c levels after accounting for other covariates in the model.

A logistic regression model was used to identify predictors of having at least one cardiovascular comorbidity (Table 10). Among the variables entered into the model, diabetes type (T2DM), age, gender, and disease duration, age emerged as the only statistically significant predictor. Specifically, each additional year of age was associated with a 12% increase in the odds of having a cardiovascular comorbidity (OR = 1.12, 95% CI [1.02–1.23], *p* = 0.019). Other predictors, including gender (OR = 0.46, *p* = 0.169), diabetes duration (OR = 0.96, *p* = 0.450), and diabetes type (OR = 0.63, *p* = 0.839), were not significantly associated with cardiovascular comorbidities in this model. Notably, while men appeared less likely than women to report cardiovascular conditions (OR < 1), this association did not reach statistical significance. Similarly, diabetes duration did not show a meaningful effect, which may reflect the influence of unmeasured confounders or the relatively small sample size.

**Figure 2:**
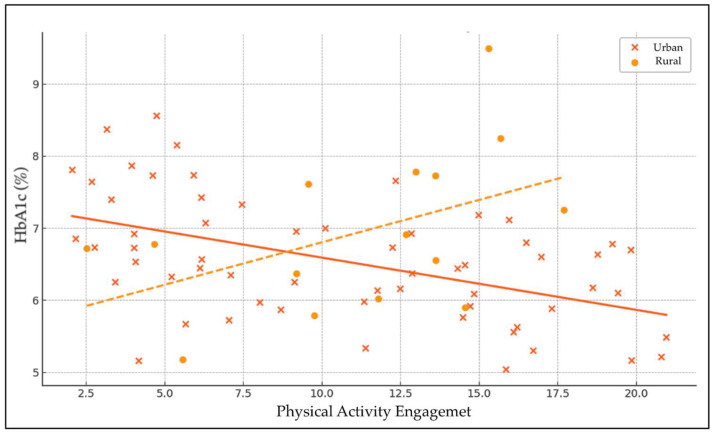
Trend-level interaction between physical-activity engagement and place of residence on HbA1c levels. Note: Fitted regression lines (shown without 95% confidence intervals for illustrative clarity) depict a non-significant trend (*p* = 0.061), suggesting a possible moderating effect of place of residence (urban vs. rural) on the association between physical-activity engagement and glycaemic control. The apparent divergence between urban and rural participants should be interpreted with caution due to the small number of rural respondents (n = 21).

The linear regression analysis examined the effects of physical activity (PA), urbanicity, and their interaction on HbA1c levels (Table 11 and Figure 2). The intercept was significant (β = 6.50, *p* < 0.001), representing the baseline HbA1c level when all predictors are zero. The main effect of physical activity on HbA1c was adverse but not statistically significant (β = −0.031, *p* = 0.370), indicating a slight, non-significant tendency for higher physical activity to be associated with lower HbA1c levels. Urbanicity alone also showed a non-significant positive association with HbA1c (β = 0.21, *p* = 0.730), suggesting no meaningful difference in glycemic control between urban and rural residents.

The interaction term between physical activity and urbanicity approached statistical significance (β = −0.081, *p* = 0.061), suggesting a potential moderating effect of urbanicity on the relationship between physical activity and HbA1c. This indicates that the relationship between physical activity and glycemic control may differ by urban or rural residence, with a trend toward greater beneficial effects of physical activity on HbA1c in one group. However, as the *p*-value slightly exceeds the conventional threshold, this finding should be interpreted with caution and warrants further investigation.

## 4. Discussion

This pilot study provides insight into the sociodemographic, clinical, and behavioral determinants of glycemic control among Croatian adults with diabetes. Higher educational attainment and participation in strength and balance training were associated with lower HbA1c levels, whereas longer disease duration and T2DM were linked to poorer glycemic control. Older age predicted the presence of cardiovascular comorbidities. No significant association was observed between overall physical activity volume or total accumulated duration and HbA1c. However, participants with better glycemic control engaged in more frequent weekly exercise and were less likely to perform short-duration sessions (<30 min), suggesting that the pattern of activity accumulation, rather than total volume alone, may be more relevant for metabolic regulation. This distinction helps clarify why total PA was not associated with HbA1c, despite group differences in how activity was distributed across the week. The lack of a direct association between PA volume and HbA1c in this cohort likely reflects both generally adequate glycaemic control among participants and the limited sensitivity of duration-based PA classifications. These findings provide preliminary evidence of behavioral and educational factors relevant to diabetes management in Croatia.

The study included 95 adults with diabetes (55.8% male, 44.2% female) with a broad age distribution. The largest subgroup comprised individuals aged 65 years and older (42.11%), followed by those aged 58–65 years (21.05%). Younger participants were less represented, reflecting the known epidemiology of diabetes, which predominantly affects older adults [39]. This age structure mirrors both national and global trends and underscores the cumulative impact of behavioral and metabolic exposures, including inactivity, obesity, and hypertension. Biological aging also contributes through mitochondrial dysfunction, reduced β-cell capacity, and increased insulin resistance. These older individuals often exhibit multimorbidity, with a higher prevalence of frailty, sarcopenia, and cardiovascular disease [40,41,42]. Such findings highlight the need for integrative interventions that combine metabolic and functional rehabilitation.

The sociodemographic profile showed a bimodal educational pattern: 56.8% had secondary education (either ongoing or completed), and 32.6% held graduate or postgraduate degrees. This distribution reflects both younger participants enrolled in education and older, professionally active individuals. Most participants were married (62.1%) and retired (50.5%), while 43.2% were employed full-time. Urban residents predominated (77.9%), consistent with healthcare accessibility patterns in Croatia, although this may obscure rural–urban disparities in preventive care and education [43].

T2DM was predominant (73.68%; n = 70), while T1DM accounted for 26.32% (n = 25). Correspondingly, 73.68% of participants received oral antidiabetic medication, and 26.32% received insulin therapy. Arterial hypertension was the most common comorbidity (63.16%; n = 60), followed by retinopathy (31.58%; n = 30), diabetic foot (15.79%; n = 15), nephropathy (12.63%; n = 12), neuropathy or polyneuropathy (8.42%; n = 8), and cardiovascular disease (12.63%; n = 12). Other chronic conditions, including autoimmune and oncological diseases, were reported by 10.53% (n = 10). These values closely align with international epidemiological data, which demonstrate a predominance of microvascular complications and highlight the clustering of hypertension, dyslipidemia, and obesity [44].

BMI analysis revealed that 28.72% of participants (n = 27) had normal weight, 42.55% (n = 40) were overweight, and 26.60% (n = 25) were obese, including 7.45% (n = 7) classified as severely obese and 2.13% (n = 2) as morbidly obese. This distribution reflects a typical pattern in outpatient diabetes cohorts, where overweight and obesity remain major contributors to insulin resistance [45,46,47]. Education and socioeconomic status likely mediate dietary quality and lifestyle behaviors [48].

Physical activity patterns revealed that 94.74% (n = 90) reported walking or brisk walking, 21.05% (n = 20) cycling, 15.79% (n = 15) swimming, 12.63% (n = 12) hiking or Nordic walking, and 10.53% (n = 10) dancing, yoga, or Pilates. Strength and resistance training were performed by 26.32% (n = 25) using free weights, 31.58% (n = 30) using elastic bands or body weight, and 21.05% (n = 20) using gym machines. Balance and coordination activities, such as one-leg stands or tai chi, were reported by 21.1% (n = 20) of participants. Regarding session duration, 26.3% (n = 25) reported exercising for less than 30 min, 52.6% (n = 50) for 30–60 min, and 21.1% (n = 20) for 60–120 min per session. Regarding frequency, 36.84% (n = 35) exercised daily, 42.11% (n = 40) at least three times per week, 15.79% (n = 15) twice per week, and 5.26% (n = 5) less than twice per week. This pattern, high walking prevalence but low participation in structured strength and balance programs, parallels global evidence suggesting that most patients prioritize accessible aerobic exercise while neglecting resistance and balance components essential for preventing frailty, sarcopenia, and falls [49,50,51].

Despite these encouraging participation rates, the study found no statistically significant association between total physical activity and HbA1c. This result may reflect a ceiling effect, given that most participants achieved satisfactory glycemic control (mean HbA1c = 6.9%). It also suggests that exercise alone may not strongly influence glycemic variability when confounded by medication adherence, diet, and disease duration. Longer disease duration (β = 0.03, *p* = 0.004) and type 2 diabetes (β = 0.38, *p* = 0.030) predicted higher HbA1c, consistent with previous research linking chronic disease exposure to metabolic deterioration [50,52,53]. The direction of the effect, showing slightly higher HbA1c among more active individuals, likely reflects reverse causality: patients with longer disease duration and greater complication burden often engage in more intensive exercise regimens but face persistent metabolic challenges [44]. Significantly, the benefits of physical activity extend beyond glycemic control, improving cardiovascular function, mobility, and mental well-being, and remain a cornerstone in obesity management, primarily through its effects on energy balance [54].

Regression analyses confirmed that education and targeted exercise independently contribute to glycemic outcomes. Higher educational attainment predicted lower HbA1c (β = –0.48, *p* = 0.013), and the inclusion of strength/balance training attenuated this effect (β = –0.32, *p* = 0.041), indicating partial mediation. Longer disease duration (β = 0.03, *p* = 0.004) and type 2 diabetes (β = 0.38, *p* = 0.030) predicted higher HbA1c, consistent with previous research linking chronic disease exposure to metabolic [50]. Logistic regression identified age as the only significant predictor of cardiovascular comorbidity (OR = 1.12, 95% CI = 1.02–1.23, *p* = 0.019). These findings align with prior evidence emphasizing aging as a dominant driver of vascular pathology in diabetes [33,41,42].

The borderline interaction between physical activity and urbanicity (*p* = 0.061) approached but did not reach statistical significance. This suggests a possible contextual effect, with exercise benefits moderated by environmental or social factors. However, this finding should be interpreted as exploratory, and confirmation would require a larger, balanced study [55].

Despite limitations deriving from the study design, particularly its pilot nature, this study provides initial evidence that educational attainment and engagement in structured exercise, particularly strength and balance training, may be relevant correlates of glycemic control. These findings align with international and national strategies that emphasize education and lifestyle modification as core components of diabetes management [3,4,56]. Tailored interventions combining patient education, individualized counseling, and progressive exercise programs could therefore enhance diabetes outcomes and functional capacity in this population. Translationally, health systems should treat education as a modifiable exposure: plain-language materials, subsidized community gyms, and professional-led balance and strength classes can function as a “social prosthesis” for those lacking formal schooling. Without such scaffolding, the socioeconomic gap in diabetes outcomes documented across Europe is unlikely to narrow [57].

### 4.1. Recommendations for Future Research and Clinical Practice

Our findings indicate that participants with a university degree or higher had lower HbA1c levels than those with lower educational attainment, suggesting that education may enhance diabetes self-management by improving health literacy, increasing access to health resources, and enhancing treatment adherence. Regular participation in strength and balance training was also associated with better glycemic control. Together, these results emphasize that modifiable factors, particularly education and structured physical activity, play a measurable role in glycemic outcomes, even after accounting for disease duration and diabetes type.

Future studies should expand on these findings by evaluating how tailored, education-sensitive interventions and functionally diverse exercise programs can be integrated into diabetes care. Such programs should combine aerobic, resistance, and balance components to target both metabolic and functional outcomes. Clinically, these findings underscore the importance of individualized management approaches that consider patients’ educational background, physical activity preferences, and contextual factors such as urban versus rural living environments. Although the observed patterns in cluster analysis suggested meaningful subgroup differences, these trends should be interpreted as exploratory and confirmed in larger, more representative samples.

### 4.2. Limitations

This pilot study has several methodological limitations. The modest sample size and cross-sectional design restrict the generalizability of the findings and preclude causal inference. Participants were recruited during routine clinical visits and may represent a more health-conscious subgroup, contributing to selection bias and the relatively low mean HbA1c observed. Physical activity was self-reported, introducing potential recall and social-desirability bias, and was assessed using session duration rather than physiological intensity. Consequently, the measure reflects duration-based activity levels rather than the WHO/CDC intensity definitions based on metabolic or exertional criteria, which may have attenuated true associations. Dietary habits and treatment adherence were not assessed with validated tools, leaving residual confounding. Because of the small sample, type 1 and type 2 diabetes were analyzed jointly to preserve statistical power, potentially masking type-specific differences. The sample was skewed toward individuals with relatively good glycaemic control, potentially limiting variability and reducing sensitivity to detect associations. Missing data were minimal (<10%) but not imputed, and the near-significant interaction between physical activity and place of residence, as well as the cluster analysis findings, should be considered exploratory and confirmed in larger, longitudinal, multicentre studies.

## 5. Conclusions

This pilot study indicates that both clinical and lifestyle factors shape glycemic control in individuals with diabetes. Higher education and engagement in strength and balance training were key predictors of lower HbA1c, whereas general physical activity showed no significant association with glycemic outcomes. Elevated HbA1c correlated with hypertension and retinopathy, emphasizing the importance of early and sustained glucose control. Older age predicted cardiovascular comorbidity, reinforcing the need for early prevention. Education may indirectly enhance metabolic outcomes through structured exercise, underscoring the value of health literacy–based interventions. However, findings should be interpreted cautiously owing to the small sample size and the duration-based rather than intensity-based measure of physical activity. Future research should include validated intensity metrics (e.g., METs) and dietary data to refine behavioral modeling.

## Figures and Tables

**Table 1:** Sociodemographic characteristics of the sample (N = 95).

Construct/Variable	Category	N (%)
gender	male	53 (55.79%)
	female	42 (44.21%)
age group	18–25	4 (4.21%)
	26–33	3 (3.16%)
	34–41	6 (6.32%)
	42–49	10 (10.53%)
	50–57	12 (12.63%)
	58–65	20 (21.05%)
	65+	40 (42.11%)
educational attainment	secondary school education (in progress or completed)	54 (56.84%)
	completed high school diploma	10 (10.53%)
	undergraduate degree completed	14 (14.74%)
	graduate degree or higher	31 (32.63%)
marital status	married	59 (62.11%)
	single (never married)	13 (13.68%)
	widowed	12 (12.63%)
	divorced	5 (5.26%)
	cohabiting partnership	1 (1.05%)
	in a relationship	1 (1.05%)
	engaged	1 (1.05%)
	not specified/other	3 (3.16%)
place of residence	urban (town/city)	74 (77.89%)
	rural (village)	21 (22.11%)
employment status	retired	48 (50.53%)
	employed, full-time	41 (43.16%)
	employed, part-time	2 (2.11%)
	unemployed	4 (4.21%)

Note: Percentages may not sum to 100% due to multiple responses and rounding.

**Table 2:** Clinical characteristics and physical activity patterns among individuals with diabetes (N = 95).

Construct/Variable	Category	N (%)
type of diabetes	T1DM	25 (26.32%)
	T2DM	70 (73.68%)
treatment type	insulin	25 (26.32%)
	oral medication (tablets)	70 (73.68%)
complications/comorbidities	arterial hypertension	60 (63.16%)
	diabetic foot	15 (15.79%)
	retinopathy	30 (31.58%)
	nephropathy	12 (12.63%)
	neuropathy/polyneuropathy	8 (8.42%)
	cardiovascular (myocardial infarction, stroke, etc.)	12 (12.63%)
	other (psoriasis, cancer, etc.)	10 (10.53%)
aerobic exercise	walking/brisk walking	90 (94.74%)
	cycling	20 (21.05%)
	swimming	15 (15.79%)
	hiking/nordic walking	12 (12.63%)
	dance/yoga/pilates	10 (10.53%)
anaerobic/strength	free weights/dumbbells	25 (26.32%)
	elastic bands/body-weight	30 (31.58%)
	gym machines	20 (21.05%)
balance/coordination	one-leg stand/tai-chi	20 (21.05%)
exercise duration	low (<30 min)	25 (26.32%)
	moderate (30–60 min)	50 (52.63%)
	high (60–120 min)	20 (21.05%)
exercise frequency	every day	35 (36.84%)
	≥3 times per week	40 (42.11%)
	2 times per week	15 (15.79%)
	<2 times per week/irregular	5 (5.26%)
body mass index (BMI)	18.5–24.99	27 (28.72%)
	25–29.99	40 (42.55%)
	30–34.99	16 (17.02%)
	35–39.99	7 (7.45%)
	≥40	2 (2.13%)

Note: Percentages may not sum to 100% due to multiple responses and rounding.

**Table 3:** Fasting glucose, HbA1c, cholesterol, and creatinine: summary statistics.

Variable	N	Mean	SD	Min	Max
fasting glucose (mmol/L)	82	7.9	2.3	4.2	16.5
HbA1c (%)	90	6.9	1.3	5.0	12.7
total cholesterol (mmol/L)	88	4.6	1.1	2.1	7.8
serum creatinine (µmol/L)	81	76.8	34.6	0.7	204.0

Note: N values vary due to missing data. The inclusion of range allows for a more nuanced interpretation of inter-individual variability within each clinical parameter.

**Table 4:** Comparison of Physical Activity Characteristics Between HbA1c Groups.

Variable	HbA1c ≤ 7.5% (n = 45)	HbA1c > 7.5% (n = 32)	Test	*p*
Weekly exercise frequency (1–5)	3.7 ± 0.8	3.1 ± 0.7	Welch’s *t*-test	0.015
Exercise session duration			χ^2^ test	
<30 min	18.0%	34.4%		0.041
30–60 min	46.7%	40.6%		0.372
60–120 min	35.3%	25.0%		0.229

Note: Welch’s *t*-test was used for unequal sample sizes and heterogeneous variances. Duration categories reflect self-reported session length.

**Table 5:** Comparison of HbA1c values between physically active and less active individuals.

Group	N	Mean HbA1c ± SD	t (df)	*p*	Cohen’s d	95% CI of the Difference
active	75	6.86 ± 1.25				
less active	20	6.67 ± 1.01	−0.72 (36)	0.479	0.16	−0.35 to 0.73

Note: Welch’s *t*-test was used due to unequal group sizes (N = 75 vs. N = 20) and heterogeneity of variances. Groups were defined by frequency-based criteria: active = exercising ≥ 3 times per week or daily; less active = exercising ≤ 2 times per week or irregularly. A duration-based (time-per-session) sensitivity definition is provided in the Methods section.

**Table 6:** Prevalence of diabetes complications by HbA1c level and associated odds ratios.

Complication	Prevalence HbA1c > 7.5%	Prevalence HbA1c ≤ 7.5%	χ^2^ (1)	*p*	OR	95% CI OR
arterial hypertension	26/32	34/63	6.79	0.009	3.70	1.34–10.21
retinopathy	15/32	15/63	5.23	0.022	2.82	1.14–6.98
nephropathy	6/32	6/63	1.64	0.201	2.19	0.65–7.45

Note: HbA1c: glycated hemoglobin; OR: odds ratio; CI: confidence interval. χ^2^ (1): Chi-square tests (df = 1) were used to assess differences in complication prevalence between patients with HbA1c > 7.5% and those with HbA1c ≤ 7.5%.

**Table 7:** Predictors of engaging in strength and balance training: full logistic regression.

Predictor	OR	95% CI	*p*
education ≥ university level	2.45	1.12–5.36	0.025
age (years)	0.97	0.94–1.01	0.110
gender (male)	1.20	0.55–2.63	0.650
duration of diabetes (years)	1.04	0.99–1.10	0.120
T2DM (vs. T1DM)	0.83	0.32–2.10	0.700
insulin therapy	1.35	0.53–3.42	0.530

Note: OR: odds ratio; CI: confidence interval. Full logistic regression model with education, age, gender, diabetes duration, diabetes type, and insulin therapy as predictors of engaging in strength and balance training at least once per week.

**Table 8:** Predictors of engaging in strength and balance training: parsimonious logistic regression Model.

Predictor	OR	95% CI for OR	*p*
constant	0.01	0.00–0.69	0.033
T2DM (vs. T1DM)	26.51	0.03–24,329.90	0.346
age (years)	1.05	0.91–1.22	0.505
male	0.87	0.16–4.72	0.875
duration of diabetes (years)	1.04	0.89–1.22	0.611

Note: Parsimonious logistic regression model including selected clinical and demographic predictors of engaging in strength or balance training at least once per week. OR: odds ratio; CI: confidence interval.

**Table 9:** Predictors of HbA1c (%): linear regression.

Predictor	β	SE	*p*
(intercept)	8.05	0.64	<0.001
education ≥ university level	−0.48	0.19	0.013
strength/balance training	−0.32	0.15	0.041
age (years)	0.01	0.01	0.080
gender (male)	−0.05	0.14	0.730
disease duration (years)	0.03	0.01	0.004
T2DM (vs. T1DM)	0.38	0.17	0.030

Note: Results from the linear regression model assessing predictors of HbA1c levels. β: unstandardized regression coefficient; SE: standard error; HbA1c: glycated hemoglobin; T2DM: type 2 diabetes mellitus (reference category = type 1 diabetes); education: dichotomized as university degree or higher vs. lower educational attainment; strength/balance training: engaging in such activity at least once per week (yes = 1, no = 0); gender: male = 1, female = 0.

**Table 10:** Predictors of cardiovascular comorbidities: logistic regression.

Predictor	OR	95% CI OR	*p*
const	0.01	0.00–0.13	0.001
T2DM	0.63	0.01–55.41	0.839
age	1.12	1.02–1.23	0.019
male	0.46	0.15–1.39	0.169
disease duration	0.96	0.87–1.06	0.450

Note: Logistic regression model assessing predictors of having ≥1 cardiovascular comorbidity (e.g., myocardial infarction, stroke, angina). OR = odds ratio; CI = confidence interval; *p* = *p*-value; T2DM = type 2 diabetes mellitus (reference category = type 1 diabetes); age and disease duration are measured in years; gender coded as 1 = male, 0 = female.

**Table 11:** Predictors of HbA1c levels: linear regression.

Coefficient	β (SE)	95% CI	*p*
intercept	6.50 (0.55)	5.41–7.59	<0.001
PA (β_1_)	–0.031 (0.034)	–0.099–0.037	0.370
urbanicity (β_2_)	0.21 (0.59)	–0.96–1.38	0.730
interaction PA × urbanicity (β_3_)	–0.081 (0.042)	–0.165–0.003	0.061

Note: Multiple linear regression analysis examining the association between physical activity (PA), urbanicity, and their interaction on glycated hemoglobin (HbA1c) levels. β = unstandardized regression coefficient; SE = standard error; 95% CI = 95% confidence interval. The interaction term tests whether the effect of physical activity on HbA1c differs between urban and rural residents.

## Data Availability

The original contributions presented in this study are included in the article. Further inquiries can be directed to the corresponding authors.

## Public Involvement Statement

No public involvement in any aspect of this research.

## Guidelines and Standards Statement

This manuscript was prepared in accordance with the STROBE (Strengthening the Reporting of Observational Studies in Epidemiology) guidelines for cross-sectional studies.

## Abbreviations

The following abbreviations are used in this manuscript:

DM	Diabetes mellitus
T1DM	Type 1 diabetes mellitus
T2DM	Type 2 diabetes mellitus
HbA1c	Hemoglobin A1c (glycated hemoglobin)
PA	Physical activity
BMI	Body mass index
GLP-1	Glucagon-like peptide-1
DPN	Diabetic peripheral neuropathy
MI	Myocardial infarction
